# Coping with the Environment: How Microbes Survive Environmental Challenges

**DOI:** 10.1155/2011/379519

**Published:** 2011-11-10

**Authors:** Haichun Gao, Tao Weitao, Qiang He

**Affiliations:** ^1^Institute of Microbiology, College of Life Sciences, Zhejiang University, Hangzhou, Zhejiang 310058, China; ^2^Department of Biology, The University of Texas at San Antonio, San Antonio, TX 78249, USA; ^3^Department of Civil and Environmental Engineering, The University of Tennessee, Knoxville, TN 37996, USA

Microorganisms are exposed to constantly changing environments in their natural habitats either in planktonic form or microbial communities. Examples of these environmental changes encompass nutrient limitation, temperature, pH, and osmolarity fluctuations, radiation, and other harmful agents, such as excessive amount of superoxides and heavy metals. To respond and adapt to adverse environmental changes, microorganisms employ a striking combination of transcriptional regulatory circuits to sense and translate extracellular stimuli into specific cellular signals, resulting in altered gene expression and protein activities. Investigation of these underlying mechanisms and strategies could improve our understanding of microbial physiology in general and potentially lead to discoveries of practical value in the field of health care and environment protection. In this special issue, we have invited a few papers that explore this topic from various perspectives. 

The first paper “*Exposure to glycolytic carbon sources reveals a novel layer of regulation for the MalT regulon*” presents the study on a synthetic lethal mutant *ompR malT^con^* of *Escherichia coli* in which the constitutively expressed maltose system regulator MalT in its active form causes cell death in the absence of the osmoregulator OmpR. The authors propose that glycolysis provides a new layer of regulation to the maltose system on the basis that addition of glycolytic carbon sources promotes viability of the mutant.

The second paper “*The effect of sub-MIC *β*-lactam antibiotic exposure of Pseudomonas aeruginosa strains from people with cystic fibrosis in a desiccation survival model*” examines resistance of *Pseudomonas aeruginosa* strains to desiccation, which is an important environmental factor to the loss of viability of cells. Strains with a mucoid phenotype exhibit significantly improved desiccation survival when compared with nonmucoid ones, but effects of preincubation with sub-MIC beta-lactam antibiotics on desiccation resistance appear to be agent specific. 

The third paper “*Detection of bacterial endospores in soil by terbium fluorescence*” of this special issue addresses the relationship of soil parameters (carbon-to-nitrogen ratio) on the occurrence of bacterial spores and distribution of spores in relation to sampling depth. The results demonstrate that the combination of microwave treatment of soil samples and measurement of terbium dipicolinate (DPA) photoluminescence is a rapid and reliable method for the assessment of bacterial spores.

The fourth paper “*The sulfate-rich and extreme saline sediment of the ephemeral Tirez lagoon: a biotope for acetoclastic sulfate-reducing bacteria and hydrogenotrophic methanogenic archaea*” is on the composition of methanogenic archaea, sulfate-reducing and sulfur-oxidizing prokaryotes in the extreme athalassohaline and particularly sulfate-rich sediment of Tirez Lagoon. The occurrence of hydrogenotrophic methanogenic and acetotrophic sulfate-reducing organisms and winter-summer community structures in Tirez sediment are analyzed by using the PCR-DGGE fingerprint technique for the functional adenosine-5′-phosphosulfate (*aprA*) and the methyl coenzyme M reductase (*mcrA*) gene markers. 

The last paper “*Stress responses of Shewanella*” of this special issue focuses on stress responses of *Shewanella*, a group of facultative anaerobes capable of respiring on an array of compounds. As important and potential microorganisms for bioremediation, stress responses of *Shewanella* have been subjected to an array of analyses, especially with high-throughput technologies such as whole-genome microarrays. This comprehensive review highlights the current understanding of mechanisms by which *Shewanella* survive and thrive within varied environments, as well as points out promising future research directions. 

**Haichun Gao** **Haichun Gao** **Tao Weitao** **Tao Weitao** **Qiang He** **Qiang He**

